# The association between APOA5 haplotypes and plasma lipids is not modified by energy or fat intake: The Czech HAPIEE study

**DOI:** 10.1016/j.numecd.2013.08.008

**Published:** 2014-03

**Authors:** J.A. Hubacek, A. Peasey, R. Kubinova, H. Pikhart, M. Bobak

**Affiliations:** a Centre for Experimental Medicine, Institute for Clinical and Experimental Medicine, Prague, Czech Republic; b Department of Epidemiology and Public Health, University College London, London, 1-19 Torrington Place, London WC1E 6BT, UK; c National Institute of Public Health, Prague, Czech Republic

**Keywords:** Apolipoprotein A5, Triglycerides, Polymorphism, Total energy intake, Interaction, APOA5, apolipoprotein A5, FFQ, food frequency questionnaire, HAPIEE, Health Alcohol and Psychosocial factors In Eastern Europe, HDL, high density lipoprotein, PCR, polymerase chain reaction, RFLP, restriction fragment length polymorphism, TG, triglycerides, VLDL, very low density lipoproteins

## Abstract

**Background and aims:**

Several smaller studies reported interactions between dietary factors and apolipoprotein A5 (*APOA5*) gene polymorphisms in determination of plasma lipids. We tested interactions between *APOA5* haplotypes and dietary intake in determination of plasma triglycerides (TG) and other lipids.

**Methods and results:**

Participants (5487 males and females aged 45–69) were classified according to the number (0, 1, 2+) of minor *APOA5* alleles (using T-1131 > C; rs662799 and Ser19 > Trp; rs3135506 polymorphisms) and into three groups of low (bottom 25%), medium (26th–75th percentile) and high (top 25%) of intake of total energy and total, saturated and polyunsaturated fats, assessed by food frequency questionnaire. The age-sex adjusted geometric means of plasma TG increased with the number of minor alleles, from 1.57 (standard error 0.01), to 1.79 (0.02) to 2.29 (0.10) mmol/L (*p* < 0.00001) but TG did not differ between groups with low, medium and high total energy intake (*p* = 0.251). TG concentrations were highest in subjects with the combination of 2+ minor alleles and the highest energy intake (mean 2.59 [0.19], compared with 1.62 [0.03] in subjects with lowest energy intake and no minor allele) but the interaction between energy intake and *APOA5* haplotypes was not statistically significant (*p* = 0.186). Analogous analyses with total, saturated and polyunsaturated fat intake yielded similar nonsignificant results. Effects of *APOA5* and dietary intakes on total and HDL cholesterol were weaker and no interactions were significant.

**Conclusion:**

In this Slavic Caucasian population sample, we did not detect the hypothesized interaction between common SNPs within the *APOA5* gene and diet in determination of blood lipids.

## Introduction

Plasma levels of triglycerides (TG) are independent risk factor of cardiovascular disease development [1]. The plasma levels of TG are significantly genetically determined. Probably the most important environmental factor that may interact with genetic polymorphisms in determination of the plasma TG levels is diet. There is growing interest in effect modification between genes and environment because such interactions could explain a number of discrepancies, such as differences in results between association studies in different populations or inconsistent effects of dietary interventions. However, the number of studies addressing gene–environment interactions on sufficient number of individuals remains modest.

The most significant impact on plasma TG levels seems to be associated with apolipoprotein A5 gene (*APOA5*, gene ID 116519, OMIM accession number 606368) variants [2], [3]. ApoA5 is located on TG-rich and high density lipoprotein (HDL) particles, enhances the activity of lipoprotein lipase [4], [5], and recombinant apoA5 binds to the LDL receptor family members [6]. Minor alleles of two tagging *APOA5* SNPs T-1131 > C [rs662799] and Ser19 > Trp [C56 > G, rs3135506] were associated, although with different strengths, with elevated plasma TG levels, regardless of ethnicity and sex [3], [7], [8], [9], [10].

Several studies explored interactions of the effects of *APOA5* variants on different biochemical traits with dietary factors. The results suggest that the *APOA5* genotypes modify the effects of dietary interventions (e.g. low/high fat diet) [11], [12], [13], [14], intake of fat [15], [16] or alcohol intake [17] on triglycerides (and less consistently on other lipids). Since previous studies have been relatively small, used different designs, selected patients and ethnically mixed populations, the results remain inconclusive.

In this study, we have investigated the potential interaction of *APOA5* with energy and fat intake in a large sample of a general Slavonic Caucasian population. Our working hypothesis was that plasma levels of TG are highest in the combination of high energy/fat intake and two or more minor alleles of *APOA5* haplotypes (based on the analysis of two polymorphisms (T-1131 > C and Ser19 > Trp).

## Methods

### Study population and study subjects

The study sample comes from the Czech part of the HAPIEE (Health, Alcohol and Psychosocial factors In Eastern Europe) project. The study examined random samples of men and women aged 45–69 years in seven Czech towns: Jihlava, Havirov, Hradec Kralove, Karvina, Kromeriz, Liberec and Usti nad Labem. Details of the study have been described elsewhere [18]. Briefly, of the 8856 individuals recruited (response rate 55%), 6681 (3079 males and 3602 females) had DNA samples available. Of these, 5847 people with non-missing data on all variables of interest are included in the analyses reported here. The subjects completed an extensive questionnaire on medical history, health status, life style, diet and socioeconomic and psychosocial factors, underwent a short examination, including anthropometry, and provided a fasting blood sample. The study was approved by the Local Ethics Committees at both Czech National Institute of Public Health and University College London, UK.

### Laboratory analyses

DNA was extracted using salting out method, and *APOA5* SNPs rs662799 and rs3135506 were genotyped using PCR – RFLP as described in details elsewhere [9]. Subjects were classified according to the presence of minor *APOA5* alleles (C-1131 and Trp19) into three groups – 0, 1, 2 and more minor alleles present.

Plasma levels of TG, total cholesterol and HDL cholesterol were analysed enzymatically using autoanalyzers and conventional methods with reagents from Boehringer Mannheim Diagnostics and Hoffmann-La Roche. The laboratory (IKEM, Prague) is accredited by CDC, Atlanta.

### Assessment of diet

Diet was assessed by a 143-item food frequency questionnaire (FFQ) with specified portion sizes adapted from FFQ previously used in the US [19] and the UK [20]. The intakes of total energy and fats (and other nutrients) were estimated from the FFQ data using the McCance and Widdowson's *The Composition of Foods* [21], with correction for differences in the composition of principal foods and adding composition of local foods and recipes [22]. For the present analyses, subjects were classified into three groups according to low (bottom 25%), medium (25th–75th percentile) and high (top 25%) intakes of total energy and total, saturated and polyunsaturated fat (as proportion of total energy); sex-specific cut-off points were used to create these categories.

### Statistical analysis

After excluding subjects with unreliable dietary data and missing data for covariates, 5847 individuals with valid *APOA5* genotype were included in the analysis. The associations of TG, total cholesterol and HDL cholesterol with energy intake and *APO5* haplotype was evaluate by linear regression for males and females separately, controlling for age. Interactions were assessed by adding interaction terms to the linear regression models. Results are reported as means and standard errors (SE); two-sided *p*-values are also given. Since the distribution of TG was skewed, TG values were logarithmically transformed. STATA statistical software (version 12; College Station, TX) was used for all statistical analyses.

## Results

Descriptive characteristics of the individuals included in the analysis are summarized in Table 1. The genotype frequencies of both polymorphisms did not differ significantly from the previously described distributions in Caucasian populations. In the entire study sample, 4322 (73.9%) subjects were carriers of the common alleles only; 1406 (24.0%) were carriers of one minor allele; and 119 (2.0%) were carriers of at least two less common alleles.

**Table 1:** Descriptive characteristics of the study sample.

	Males	Females	All
*N*	2690	3157	5847
Age (years), mean (SE)	58.0 (6.9)	57.2 (6.9)	57.6 (7.0)
TG (mmol/L), mean (SE)	2.14 (1.65)	1.70 (0.96)	1.90 (1.34)
TC (mmol/L), mean (SE)	5.61 (1.04)	5.84 (1.03)	5.73 (1.04)
HDL-C (mmol/L), mean (SE)	1.25 (0.34)	1.51 (0.40)	1.39 (0.39)
BMI, (kg/m^2^), mean (SE)	28.2 (3.9)	28.1 (5.0)	28.2 (4.6)
Energy intake (kcal/day), mean (SE)	2096 (698)	1979 (709)	2033 (706)
Total fat (% of energy), mean (SE)	36.4 (0.1)	36.2 (0.2)	36.3 (0.1)
Saturated fats (% of energy), mean (SE)	13.1 (0.05)	13.0 (0.04)	13.1 (0.03)
Polyunsaturated fats (% of energy), mean (SE)	6.5 (0.03)	6.7 (0.03)	6.6 (0.02)
APOA5 minor alleles, *n* (%)			
0	1999 (74.3)	2323 (73.6)	4322 (73.9)
1	636 (23.6)	770 (24.4)	1406 (24.1)
2 and more	55 (2.0)	64 (2.0)	119 (2.0)

As expected, both variants had a significant effect on plasma TG levels (results not shown). When the two variants were combined into one variable indicating the number of minor alleles, the geometric means of TG increased with the number of minor *APOA5* alleles, from 1.57 (SE 0.01) mmo/L over 1.79 (0.02) mmo/L to 2.29 (0.10) mmo/L, *p* < 0.00001 (Table 2). Total cholesterol (*p* < 0.001) increased linearly and HDL-cholesterol values decreased (*p* < 0.001) with the number of minor *APOA5* alleles, and intakes of energy and fats were not associated with the number of the *APOA5* minor alleles (Table 2).

**Table 2:** Means (standard errors) of lipid parameters (in mmol/L), BMI (in kg/m^2^) and intake of energy (in kcal) and fat (as % of energy) with APOA5 haplotype, adjusted for age and sex.

	Number of the APOA5 minor alleles			*p*
	0	1	2 And more	
*N*	4322	1406	119	
Triglycerides^a^	1.57 (0.01)	1.79 (0.02)	2.29 (0.10)	0.00001
Total cholesterol	5.70 (0.07)	5.79 (0.03)	6.01 (0.09)	0.001
HDL-cholesterol	1.40 (0.01)	1.36 (0.01)	1.32 (0.03)	0.001
BMI	28.2 (0.07)	28.2 (0.12)	28.0 (0.41)	0.892
Energy intake	2036 (11)	2013 (19)	2083 (64)	0.545
Total fat	36.3 (0.1)	36.2 (0.2)	36.7 (0.6)	0.906
Saturated fats	13.1 (0.04)	13.0 (0.06)	13.2 (0.22)	0.499
Polyunsaturated fats	6.6 (0.02)	6.6 (0.04)	6.7 (0.16)	0.350

a Geometric mean.

**Figure 1:**
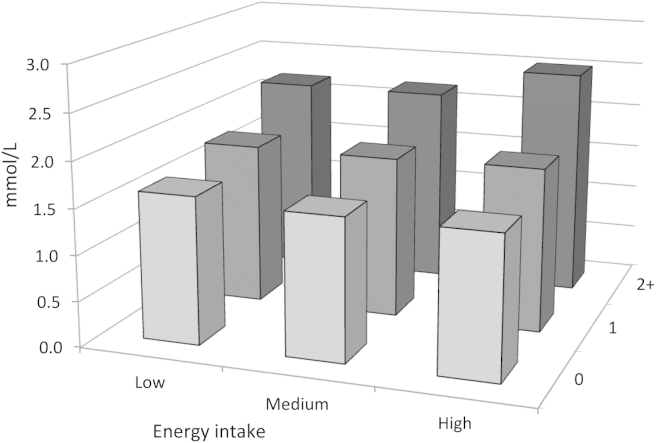
Geometric means of TG by the combination of energy intake and the number of *APOA5* minor alleles.

Plasma TG levels did not differ significantly between groups with low, medium and high total energy intake; the geometric means were 1.66 (0.02), 1.62 (0.02) and 1.63 (0.02), respectively, *p* for trend 0.251. There were no differences in lipids by intakes of total fat, saturated fat or polyunsaturated fat (not shown in table). The geometric means of TG by the combination of energy intake category and the number of minor alleles of *APOA5* are shown in Table 3. There is a suggestion that the combination of high energy intake and 2 or more minor alleles produces the highest TG levels (Fig. 1) but the interaction between total energy intake and *APOA5* haplotypes was not statistically significant (*p* = 0.186).

**Table 3:** Mean concentrations (SE) of triglycerides and total and HDL cholesterol (all in mmol/L) with the number of APOA5 minor alleles by total energy intake category, adjusted for age and sex.

Energy intake	Number of *APOA5* minor alleles			*p* for trend^a^
	0	1	2 And more	
TG				
<25%	1.62 (0.03)	1.78 (0.05)	2.19 (0.21)	<0.001
>25–75%	1.56 (0.02)	1.78 (0.03)	2.19 (0.14)	<0.001
>75%	1.55 (0.02)	1.80 (0.05)	2.52 (0.19)	<0.001
*p* for interaction^b^ = 0.186				

TC				
<25%	5.76 (0.03)	5.70 (0.06)	6.02 (0.21)	0.843
>25–75%	5.68 (0.02)	5.81 (0.04)	5.92 (0.14)	0.003
>75%	5.71 (0.03)	5.84 (0.06)	6.12 (0.17)	0.004
*p* for interaction^b^ = 0.090				

HDL				
<25%	1.41(0.01)	1.36 (0.02)	1.34 (0.07)	0.033
>25%–75%	1.40(0.01)	1.36 (0.01)	1.35 (0.05)	0.003
>75%	1.41(0.01)	1.37 (0.02)	1.22 (0.06)	0.004
*p* for interaction^b^ = 0.401				

a *p*-value for trend by the number of APOA5 minor alleles.

b *p*-values for differences in slope between 0 and 2 and more minor alleles (interaction between energy intake and APOA5).

Similarly, interactions between total energy intake and *APOA5* haplotype were not significant in determination of concentrations of total and HDL cholesterol (Table 3).

We also examined interactions with dietary intakes of total fat, saturated fat or polyunsaturated fat. None of the fat intake variables acted as effect modifiers of the association between *APOA5* haplotypes and plasma lipids (all *p* vales > 0.3, detailed results available on request). Finally, there were no interactions between dietary intakes and the individual *APOA5* polymorphisms.

We conducted additional analyses using other metabolic syndrome variables: systolic and diastolic blood pressure and blood glucose. While all these variables were associated with TG as expected (all *p*-values <0.001), none of them was significantly associated with the *APOEA5* haplotype (all *p*-values >0.4), and stratification for dietary intake of energy or fat did not identify any association with *APOA5* in any subgroup (not shown in table).

## Discussion

This study examined the potential interaction between *APOA5* haplotypes and dietary intake of energy and fats in a large general Slavic Caucasian population sample in relation to plasma lipids. We found that lipids were associated with both individual polymorphisms in *APOA5* and with their combination but not with dietary intakes of energy or saturated, unsaturated or polyunsaturated fats. Although there was a suggestion that TG are highest in carriers of at least two minor *APOA5* alleles who also had high energy intake, the interaction between *APOA5* haplotype and any of the dietary indicators were not statistically significant.

### Limitations and strengths

The common/main problem of nutrigenetic studies is insufficient statistical power. The combination of small sample size, heterogeneity of dietary assessments' and measurement inaccuracies reduce the ability of nutrigenetic studies to reliably detect interactions between genetic and dietary factors. Despite the large sample size, only 2% of subjects in our study had 2 or more minor *APOA5* alleles; this would allow detection of only relatively strong effect modifications. This may be one of the reasons why none of the tested interactions in this study were significant.

Second, as mentioned above, most methods of dietary assessment, including food frequency questionnaires, are inaccurate. This misclassification would further reduce the ability of the study to detect main effects and interactions. We have attempted to minimise this issue by comparing groups with contrasting intakes (highest and bottom 25%) but this may have not been sufficient to completely overcome the measurement bias.

Third, the subjects in this study were relatively old, and the negative findings may not be generalised to younger subjects with different life style than older persons.

However, the study also has several strengths. The study was perhaps the largest so far published on the potential *APOA5*-nutrition interaction and lipids. Second, we have combined two tagging polymorphisms to create a variable with a strong effect on plasma TG levels. Both these aspects would increase the probability of detecting an interaction with diet, if it exists. Finally, this study examined a general population sample, unselected by medical history, and the study population is ethnically homogenous.

### Interpretation

The need for the nutrigenetic analyses is widely acknowledged by numerous authors [23], [24], [25]. However, studies with sufficient numbers of well phenotypically characterised individuals and sufficient power remain scarce. So far, there is some, but limited, evidence that variants within the *APOA5* gene may interact with environmental, mainly dietary, factors.

Several experimental studies assessed whether changes in blood lipids depended on the combination of diet and *APOA5* genotype. In a dietary trial in Korean patients with hypertriglyceridemia, *APOA5* T-1131T homozygotes showed larger decrease in plasma TG after combined dietary and exercise intervention [11]. In a Chinese study, the changes in TG after high carbohydrate/low fat diet interact with *APOA5*-1131 TT genotype in women but not in men [12]. Another Koran study of low/high fat diet suggested that postprandial TG elevation differed by *APOA5* T-1131T genotype [13]. In the Pounds Lost trial, another *APOA5* variant, rs964184, influences reduction in total and LDL cholesterol in the low fat intake group [14].

In addition to experimental studies, there have also been studies in general population samples. Among 117 Czech males whose dietary composition markedly changed over an 8-year period, decrease in total cholesterol (but not TG) was associated with the Ser19 > Trp polymorphism [26], [27]. Results from the Framingham Heart study have shown that individuals consuming the diet rich in polyunsaturated fatty acids had higher fasting TG levels, if they carried at least one C-1131 allele; this interaction was specific for the omega-6 fatty acids only [15]. Interestingly, the second common *APOA5* variant (Ser19 > Trp) did not interact with high polyunsaturated fat intake. In the Boston Puerto Rican Health Study, carriers of the C-1131 allele had higher levels of TG and total cholesterol if they also had high total fat intake [16]. By contrast, no gene–diet interaction was observed in as study of 250 elderly Brazilian women in Ref. [28]. Finally, a study of a Spanish population sample reported an interaction between *APOA5*-1131, fat intake and TG but the lowest TG levels were found in the combination of minor allele with high fat intake [29]. Among studies focused on the same polymorphism as this investigation, all studies, except Sanchez-Moreno et al., found highest TG levels in subjects with the minor allele and high fat intake. This general pattern is consistent with our findings, but in our study, despite being several times larger than the others, the interaction did not reach statistical significance.

Overall, the non-significant tendency of TG being highest among subjects with the combination 2+ minor alleles and the highest energy intake, together with studies reported in the literature may be consistent with the hypothesis that common SNPs within the *APOA5* gene interact with diet in determination of blood lipids. However, the interaction is likely to be weak and a conclusive study would require a very large sample size to confirm or reject this hypothesis.
